# Supplementary material for: Self-reported voice problems in call center employees during the COVID-19 pandemic: prevalence, risk factors, and occupational conditions
Source: PeerJ. 2025 Jun 26;13:e19595. doi: 10.7717/peerj.19595 (PMC12206399; doi:10.7717/peerj.19595)
Supplement: Supplemental Information 1 [file peerj-13-19595-s001.docx]

| **Meslekteki yıl** | 0-1 | 1-3 | 3-5 | 5 ve üzeri |
| --- | --- | --- | --- | --- |
| **Günlük çalışma saati** | 0-6 | 6-8 | 8 ve üzeri |  |
| **Çalıştığınız ortamda kendinizi stres altında hissediyor musunuz** ? | evet | hayır |  |  |
| **Günlük ortalama kaç saat sesinizi aktif olarak kullanıyorsunuz** ? | 0-1 | 1-3 | 3-5 | 5 ve üzeri |
| **Su tüketme alışkanlığınızı nasıl tanımlarsınız** ? | Günde 0-1 litre | Günde 1-2 lt | Günde 2-3 lt | Günde 3 lt üzeri |
| **Konuşurken vücudunuzun duruşuna önem verir misiniz ?** | Evet | Hayır |  |  |
| **Sesin doğru kullanımı hakkında bilginiz var mı ?** | Evet | Hayır |  |  |
| **Sigara kullanıyor musunuz ?** | Evet | Hayır |  |  |
| **Çağrı merkezi çalışanı olarak işe başladığınız zamandan şu ana kadara hiç ses problemi yaşadınız mı?** | Evet | Hayır |  |  |
| **Gün içinde boğazda ağrı veya tahriş hissi şikayetiniz oluyor mu?** | Evet | Hayır |  |  |
| **Doktor tarafından tanı konulmuş gastroözafagial reflünüz var mı** ? | Evet | Hayır |  |  |
| **Solunum yollarını ilgilendirecek bilinen bir allerjiniz var mı**? | Evet | Hayır |  |  |
