# Supplementary figures and images for: Self-reported voice problems in call center employees during the COVID-19 pandemic: prevalence, risk factors, and occupational conditions
Source: PeerJ. 2025 Jun 26;13:e19595. doi: 10.7717/peerj.19595 (PMC12206399; doi:10.7717/peerj.19595)

**
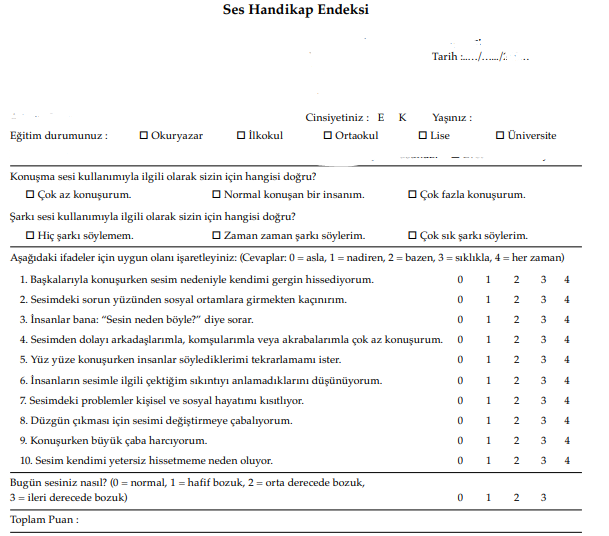
**

Supplement: Supplemental Information 2 [file peerj-13-19595-s002.docx]
